# Dental Notes

**Published:** 1899-08

**Authors:** F. S. Casper

**Affiliations:** Austin, Texas


### Dental Notes.

BY F. S. CASPER, D. D. S., AUSTIN, TEXAS.

AN UNUSUAL BEQUEST:—J. C. McKee, late of Butler, Penn., a retired lieutenant-colonel and surgeon of the United States army, made a bequest, which is so novel and unusual that we here reproduce it. Every dentist who reads this will agree with Col. McKee on the importance of the matter:

"I give and bequeath to the board of school directors of the borough of Butler, Pa., for the use of the school district of said borough, the sum of one thousand dollars ($1000) ; provided, the said school board shall, by resolution entered on its minutes, accept the same, upon condition that the said board shall require and direct the several teachers and instructors employed by them in the schools of said district from time to time, to give their pupils instructions at stated times during the school term of each year successively after my death, on the importance, as a matter of health, of the care, attention and preservation of their teeth, of the beneficial effects of the use of the tooth brush as a means of cleansing and preserving the same. And if ailing or decaying, of the importance of their timely treatment by a competent dentist, and especially to impress on their minds the penalties, which will undoubtedly follow their neglect to give attention to these personal matters, such as toothache, early loss of the teeth, *ill* appearance, bad breath, and perhaps a poisoned system, and the disgust and contempt of friends that may follow and annoy them throughout life; and that the teachers and instructors so employed shall also be required to report from time to time to the board any pupil whose teeth require treatment, but who, from poverty or other cause, neglects the same; and I request the said board to have my reasons for making this request set out on a typewritten paper accompanying this, my last will and testament, published in the newspapers of the borough of Butler on the acceptance hereof. And also on condition that the said board shall devote and use a sum of not less than fifty dollars ($50) each year, if that amount shall be necessary, in the employment of a competent dentist in said borough to treat the teeth of said pupils of said schools as shall be recommended to him by said board by certificate in writing, and whom the board may determine on application or report, of teachers or instructors, irrespective of *race, color,* politics or religion, are in need of such treatment, always preferring those whose parents or guardians are least able to pay for such treatment."

In a codicil he says: "It is my will and desire to increase my gift for the preservation of the teeth of the poor children of the common schools of the borough of Butler, Pa., mentioned in 'Item 15, letter C,' one thousand dollars, and I hereby give and bequeath one thousand dollars additional to said object, making two thousand dollars in all."—*Piscataqua Observer.*

---

Removal of an Inlay Matrix.—After carefully burnishing gold foil No. 40, into the cavity prepared for the inlay, trim off the excess of foil until the edge is even with the margins of the cavity, then fill the gold matrix thus formed with a stiff wax, flush with the margins; it can then be removed from the cavity with a sharp pointed excavator without endangering the periphery. Now, carefully invest in powdered asbestos and plaster Paris; after investing and giving sufficient time to harden, wash out the wax with boiling water, when the matrix is ready to receive the inlay body.

---

Fever Blisters.—"These troublesome little ulcers, which often interfere with dental operations, especially when located in the corners of the mouth, are promptly cured by the application of resinol ointment. W."—*Dental Office and Laboratory.*

---

Mechanical Notes.—"We have often found that perfectly clean 22-karat scraps, when melted and made to boil with heat from the blow-pipe on a piece of charcoal would crack badly when hammered on the anvil. The effect of corrosive sublimate would remove any lead, tin or zinc which may have found its way among the scraps, while saltpetre would remove any trace of iron. Our plan is not to melt the scraps on charcoal, but on a piece of an old crucible or a piece of slab, such as is used to bake artificial teeth on, which may be readily procured from any artificial tooth manufactory. Place the slab or piece of crucible in your asbestos holder, for fear the nugget might roll off when melted. As soon as it is melted take the face of a hammer and flatten the melted nugget. Done in this way the gold will rarely crack, either in rolling or hammering, while if melted on charcoal it will almost invariably crack."—*Dental Office and Laboratory.*

The editors of *The Dental Digest* and the *International Dental Journal,* two of our most welcome exchanges, whose writings are considered authority upon all dental questions, we are sorry to see, have buckled on their editorial armor and crossed swords over the question of the overproduction of the dental colleges. In other words, there are too many recruits to the ranks of an already overcrowded profession. Dr. Crouse of the *The Digest,* claims, however, that too many students are admitted to the colleges who are not suited by nature to follow the calling of dentistry, and that they find their error out when they have spent their money and lost several years of their valuable time, which should have been employed in a calling more suited to their nature. Dr. Truman takes exception to the assertion, as he is the dean of the dental department of the University of Pennsylvania, he seems to think it a shot at him. We do not think that Dr. Crouse intended to convey the idea that this was a rule with the colleges; but that it often happened. We are not troubled with the overproduction of the college graduates in this "neck of the woods" as much as from the spontaneous or pseudo dentist.—Ed.

